# A Curriculum for Achieving Universal Health Care: A Case Study of Ateneo de Zamboanga University School of Medicine

**DOI:** 10.3389/fpubh.2021.612035

**Published:** 2021-04-29

**Authors:** Monserrat Guignona, Servando Halili, Fortunato Cristobal, Torres Woolley, Carole Reeve, Simone Jacquelyn Ross, André-Jacques Neusy

**Affiliations:** ^1^School of Medicine, Ateneo de Zamboanga University, Zamboanga, Philippines; ^2^Research and Extension, Zamboanga State College of Marine Sciences and Technology, Zamboanga, Philippines; ^3^College of Medicine and Dentistry, James Cook University, Townsville, QLD, Australia; ^4^School of Medicine, Flinders University, Alice Springs, NT, Australia; ^5^The Training for Health Equity Network, New York, NY, United States

**Keywords:** education, socially-accountable, curriculum, health workforce, medical

## Abstract

**Introduction:** Universal Health Care requires equal distribution of a health workforce equipped with competencies appropriate for local population needs. While health inequities persist in the Philippines, the Ateneo de Zamboanga University School of Medicine (ADZU-SOM) in Zamboanga Peninsula – an impoverished and underserved region – has demonstrated significant success retaining graduates and improving local health statistics. This study describes the qualitative evidence of ADZU-SOM students and graduates having positive impacts on local health services and communities, and the contextual factors associated with the school's socially-accountable mission and curriculum that contribute to these impacts.

**Methods:** This qualitative study involved 41 one-on-one or group interviews conducted across seven participant groups (faculty, graduates, final-year students, health professionals, health workers, community members, community leaders). Gale et al's method for analyzing qualitative data in multi-disciplinary health research, WHO's “6 Building Blocks for quality health systems” framework and THEnet's social-accountability framework were used to organize and interpret data.

**Results:** Local community members, community leaders, and health staff consistently reported examples of ADZU-SOM students and graduate doctors developing health infrastructure and providing health education, health promotion, and disease prevention activities accessible to all population groups. Students and graduates suggested these impacts were due to a number of factors, including how ADZU-SOM's sandwich model of longitudinal community-engagement culminating in 10-months continuous community placement in the final year helped them develop a strong motivation for community service, the teachings and curriculum activities that focused on public health and the social determinants of health, and faculty's commitment and ability to operationalize ADZU-SOM's mission and values. Staff also reported impacts were driven by integration of regional and national health priorities as core curriculum, and involving local stakeholders in curriculum development.

**Conclusions:** This study provides qualitative evidence that ADZU-SOM's curriculum content and immersive community placements are training a medical workforce that is strengthening local health systems and health infrastructure across all 6 WHO “Building Blocks for quality health systems.” These findings suggest ADZU-SOM has managed to evolve a consciousness toward community service among final year students and graduates, adding evidence to the assertion it is a fully socially-accountable health professions institution.

## Introduction

The health workforce is the foundation of the health care system. While the World Health Organisation (WHO) estimates that an additional 18 million doctors, nurses and midwives are needed worldwide by 2030 to achieve universal health care (UHC), too few health practitioners currently practice where they are needed most (1). Increasing the total numbers of health workers is not sufficient; they need to be equitably distributed, possess the required competencies to address relevant local health needs, and be motivated and empowered to deliver quality care that is appropriate and acceptable to the sociocultural needs of the population (2). There is also mounting evidence that the health systems in which these workers practise must also deliver services equitably and efficiently if the health status of all population groups are to be improved; taking into account the additional core components of service delivery, health information systems, access to essential medicines, financing, and leadership/governance (3).

Workforce shortage, skill-mix imbalances, and maldistribution of human resource for health are some identified barriers to the successful implementation of UHC. Contributing to this barriers is the failure of Health Professional Education to adjust medical education to the changing conditions of the healthcare delivery system because of curricular rigidities, professional silos, static pedagogies, and insufficient adaptation to the local context (4).

Socially accountable health professional education (SAHPE) aims to address this workforce maldistribution and greater accessibility of health services in general through increasing the quantity (including distribution and retention), quality, and relevance of health care providers to their communities. “The WHO believes the social mission of health professional institutions should represent an opportunity to nurture public service ethics, professional values and social accountability attitudes required to deliver care that responds to community needs and population expectations” (2). The Global Consensus on social responsibility states that the added value of socially accountable schools is their commitment to ensuring that their students, graduates, research activities, and health care models improve the health status across all community population groups (5). For this to be achieved, the health curricula must address local needs and be grounded in competency-based learning, including how to engage effectively with local communities to address the social determinants of health (5). However, these are relatively new concepts. Evidence is required on what works, how and in what context, as very few studies in the literature look at the impact of this approach (6).

The Philippines suffers significantly from health inequities, with rural areas having poorer health and less health workforce (especially doctors) compared to urban areas. Although, child mortality is declining overall across the Philippines – a key indicator of health status – inequities in distribution across different economic strata is getting wider in rural locations (7). In addition to the urban-rural maldistribution of health professionals, almost 70% of Philippine doctors and more than 80% of nurses end up practicing overseas, further draining the health workforce (8). The medical education system in the Philippines is also greatly influenced by the western curricula. The system is discipline-based, teacher centered, and classroom lecture is the main venue and method of delivering teaching-learning activities. The focus primarily is on the disease and hospital-based individual care, resulting in a mismatch between competencies gained by health professionals and the needs of the population. This further leads to mismanagement of human health resources and reduced access to the healthcare system (9). However, against this national trend, the socially-accountable ADZU-SOM has had great success in graduate retention rates and distribution in local areas of need.

The ADZU-SOM was founded on a mission to serve the poorest and most isolated communities in Zamboanga Peninsula. The combined degree of Medicine and Public Health (MD-MPH) curriculum goal is to train physicians with the skills and priorities oriented toward social determinants of health, intersectoral collaboration, and community participation. The teaching-learning activities are structured around the local, regional, and national health priorities. The 12 impact programs of the Philippine Department of Health became the core content of its curriculum. During the first 3 years of the medical program, students spend 1 month at the end of every semester living and learning how to practice medicine in the community. In the final year, the entire year is spent in the rural community. The students learn to use multi-sectoral collaboration and participatory approach to strengthen community capacity for health care development. While in the rural communities, students, community members and health authorities collaboratively design and implement health programs relevant to the community, as well as implement interventional research to solve real health needs. The post graduate internship is also unique; while conventional Philippines medical curriculums allot 90% of the entire internship in the hospital, ADZU-SOM students spend half the year in broader community exposure at rural health clinics, community hospitals and by undertaking community work.

Recent studies found more than 90% of ADZU-SOM students work in local Philippines communities (10); often practising in areas that never previously have had a doctor. This has resulted in a 55% increase in the number of Zamboanga Peninsula municipalities having a medical practitioner over the last two decades (8). In addition, ADZU-SOM's social accountability philosophy has been found to impact the practice choices of its graduates (11) and strengthen community health services across the Zamboanga Peninsula (12). Further, since the establishment of ADZU-SOM in 1994, the infant mortality rate in the region has decreased by approximately 90%, compared with a national change of only 50% in the same time period (8).

With the quantitative evidence of ADZU-SOM impacts on local communities and health systems being published previously, the aim of this case study is to describe the qualitative evidence of ADZU-SOM students and graduates having a positive health impact on local health services and communities, and the contextual factors associated with the school's socially-accountable mission and curriculum that contribute to these positive outcomes. This study is part of a series of multi-institutional collaborative research carried out by THEnet and its institutional partners to gather evidence on the outcomes and impacts of socially accountable transformative health professional education, and part of a larger THEnet project and framework to build evidence on how to produce a fit-for-purpose heath workforce (13, 14).

### Setting: ADZU-SOM's History, Selection Processes, Curriculum, and Community Engagement Processes

Established in 1994, ADZU-SOM is a private, not-for-profit health professional institution with a mission to help provide solutions to the health problems of the people and communities of Zamboanga Peninsula, a remote region of the Philippines. During its establishment, ADZU-SOM was the only medical school in a region home to the 14 poorest municipalities in the country. Zamboanga Peninsula had a population of 3.4 million with 70% of the people living in rural areas, with two local provinces ranking 1st and 2nd in number of children with malnutrition and hunger, and an overall regional infant mortality of 75/1,000 livebirths (15). In addition, the average physician to population ratio was at 1:7,000 which was way below ideal, leaving 80% of the area doctorless and nearly 100 municipalities lacking medical attention (15).

ADZU-SOM has led the way in social accountability even before the term became more widely known (15). As a corollary, its establishment has made medical education more accessible to many prospective doctors who otherwise would not have the means to undertake medical education. Prior to its establishment, prospective students had to travel to other parts of the Philippines to pursue a degree in medicine, and frequently did not return.

In order to fully address the shortage of physicians across Zamboanga Peninsula, ADZU-SOM adopted a strong focus on social accountability, and in 2009, became a founding partner of the Training for Health Equity Network (THEnet) (www.thenetcommunity.org). Social accountability has been defined by Boelen and Heck (16) as: “The social obligation to direct education, research and service activities toward addressing the priority health concerns of the community, region, and/or nation the school has a mandate to serve. The priority health concerns are identified jointly by governments, health care organizations, health professionals, and the communities.” Two key ADZU-SOM priorities that align with socially-accountable principles are a need to train doctors in the region in order to retain them, and to decrease the region's high infant mortality rate.

The student selection criteria prioritize equitable access for ethnic, socioeconomic, gender, and religious groups to ensure the local population is proportionally represented, and the students selected have a desire to serve their community. The selection process also includes a non-medical person (usually from the community) to provide a community perspective. Selection criteria for scholarships place greater weight on area of origin and family income than academic success. Socioeconomically disadvantaged students and those coming from remote/rural areas are given priority for scholarships. Approximately 30% of the students become scholarship holders.

**Figure 1:**
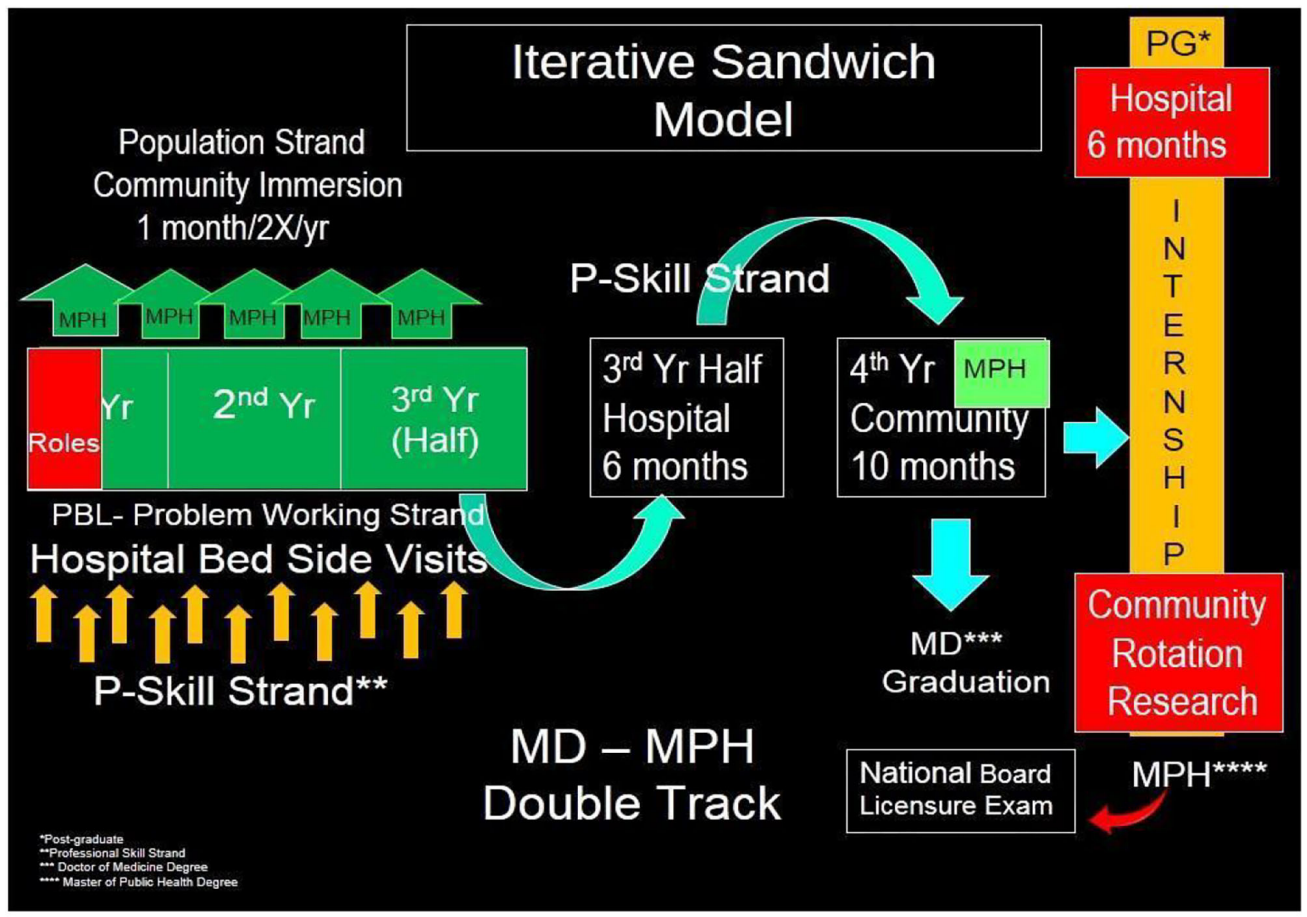
ADZU-SOM's iterative “sandwich” model for hospital- and community-based clinical rotations.

**Figure 2:**
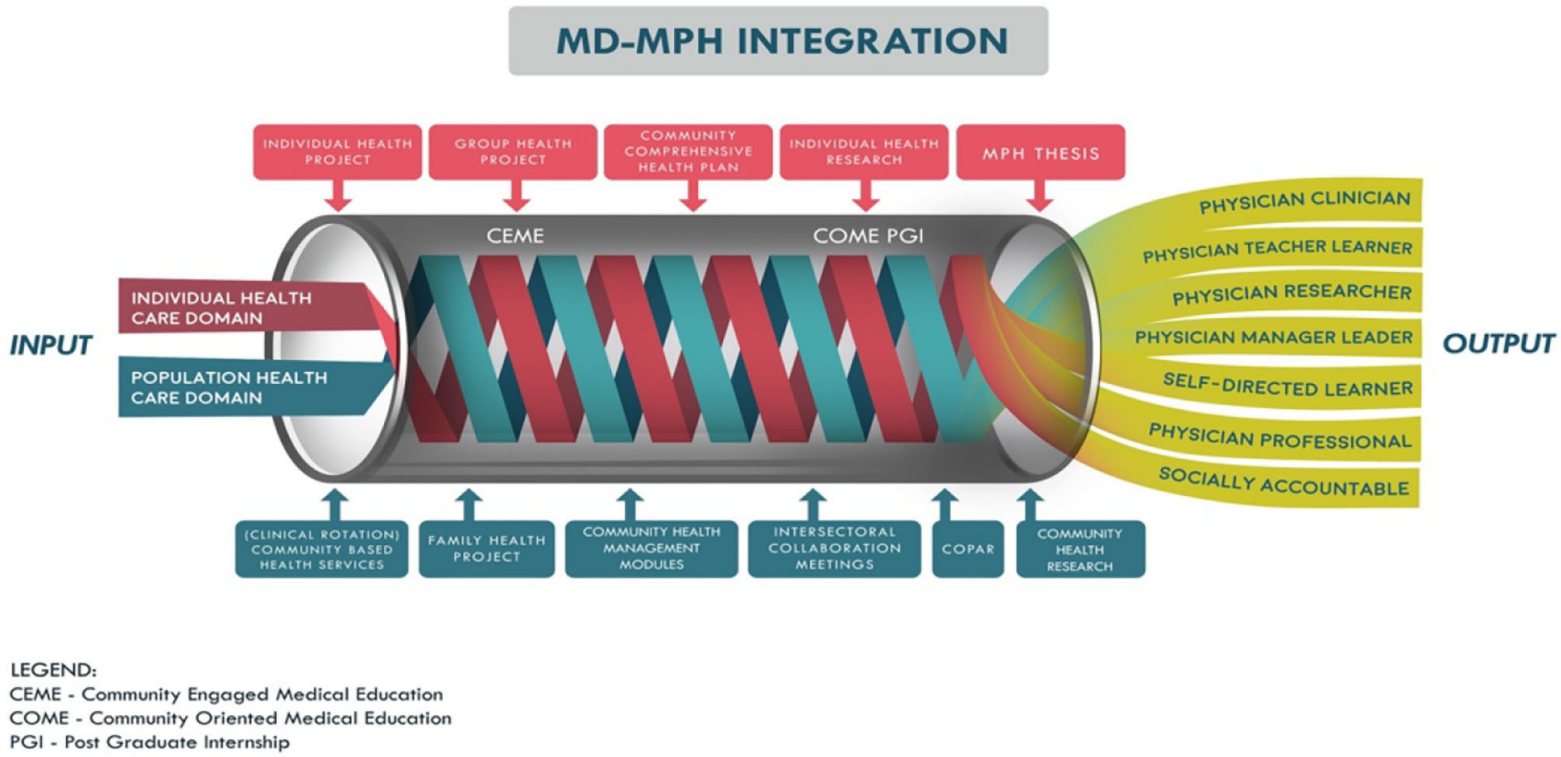
ADZU-SOM's combined MD-MPH program.

The school was founded on a mission to provide solutions to the pressing health priorities of the people and communities of Zamboanga Peninsula; therefore, the local, regional, and national health priorities became the core curriculum and the focus of student learning. Given the region's needs, ADZU-SOM's curricular design is aimed to train physicians with the competencies oriented toward social determinants of health, intersectoral collaboration, and community participation. ADZU-SOM has a four-year postgraduate program, with competencies taught including: physician manager leader, physician clinician, physician teacher, physician researcher, and physician learner, physician professional, and socially accountable physician. Instructional activities are iteratively undertaken in the university, hospitals, private clinics, patient bedsides, and community posts. Total contact hours with patients are equally divided – half in clinics and university classroom settings with a problem-based learning model, and the other half allocated for learning and serving in community settings (refer Figure 1). Another unique feature of medical education at ADZU-SOM is the double degree offered—a degree in MD-MPH. This unique feature incorporates the learning process of the individual healthcare domain and the population healthcare domain of medicine into the curriculum (refer Figure 2).

Fully-immersive, community-based service learning is a particular focus. During their short immersions in Years 1 to 3 (1 month per semester), students must develop a Community Health Plan (CHP) in collaboration with key stakeholders from a selected local community. In the initial immersion, students undertake a community survey together with community health volunteers (as there are no existing community data), then present the survey results to local community leaders. In collaboration with the community leaders, they prioritize the problems of the community to develop a fully detailed CHP. Then, during the 10-month community immersion experience in the 4th (final) year, the students fully implement this CHP in the selected community through intersectoral collaboration. Furthermore, students are required to implement individual interventional research projects aimed at providing solutions to the pressing health needs specific to the community they serve. By graduation, the CHP and the individual research project have both been completed, and the health problem should be resolving.

Community-based health research is also an indispensable part of ADZU-SOM's training of future physicians. Students are required to conduct not only research in an area of interest, but also undertake interventional studies to address problems in the community where they are assigned. These interventional studies not only contribute to the body of health knowledge but also improve the health status of the area. As of 2019, a total of 550 community-based health research projects have been completed by students, dealing with key issues like waste segregation/environmental sanitation, maternal and child health, culture and infectious disease – with all projects focusing on the national top 10 causes of morbidity and mortality.

## Methodology

### Study Design

Case study methodology was applied to enable an understanding of how and why the complex social phenomena of the SAHPE approach was effective, and to explore the impact of context on outcomes (17). Specifically, this case study links the training program and its implementation with program effects to explore the key factors contributing to social accountability for health professional schools, and to identify if ADZU-SOM students and graduates are having a noticeable impact on local health services and communities. Ethical approval for the study was obtained from the Ateneo de Zamboanga School of Medicine Ethics Review Committee and the Flinders University Human Research Ethics Committee (#7042) in Australia.

This study used a basic qualitative descriptive design (18) as the preferred qualitative method, given the desired outcome was the production of straight-forward descriptive summaries of outcomes and activities - who were involved, what was involved and where activities and experiences took place. The seven stakeholder groups used in the purposive sampling strategy were chosen to obtain the range of cases deemed most rich in information for the purpose of achieving data saturation (19).

To improve construct validity, THEnet's social accountability Framework (13, 14), developed from the cumulative experiences of THEnet founding schools, guided the development of interview questions to provide a range of evidence showing participants' described impacts and if these impacts could be attributable to aspects of ADZU-SOM's curriculum “intervention.”

### Data Collection

Evidence of SAHPE outcomes and impacts was assessed *via* 41 interviews and/or focus groups across seven participant groups: academic/faculty (3 interviews), graduates (10 interviews), final year students (2 focus groups, 10 students), health professionals (5 interviews), community health workers (5 interviews), community members (5 interviews), and community leaders (3 interviews).

Using the THEnet Framework for social accountability (13, 14), the focus group/interview questions were developed around three key areas: (1) school social accountability values; (2) curriculum and faculty; and (3) community health services and needs. The questions were refined by the researchers through group discussion, and a comprehensive interview guide created to standardise the process of administering the questions across participant groups. A written consent was obtained per informant prior to the start of data collection.

The final year students were selected randomly from student lists, while the other participants were purposefully chosen to provide critical reflection and/or with corporate knowledge of the school. Participation was voluntary, with personal identifiable information replaced by codes to assure participant confidentiality and anonymity. A fellow student facilitated the focus group discussions to minimise the potential risk of students feeling vulnerable when sharing their experiences with academic staff.

The questions were piloted with the respective participants, and then modified for clarity and cultural appropriateness. The questions for community members were further translated into local dialects. Three rounds of interviews and/or focus groups occurred. After the first round of interviews, modifications led to questions being more open-ended and tailored to specific participant groups. A second round of interviews continued until data saturation was reached; that is, no new themes were identified (19). To enhance trustworthiness, a preliminary analysis of the data was undertaken to identify key concepts, then a third round of interviews asked more in-depth probing questions to confirm and further explore key findings as per “member checking” recommended by Lincoln and Guba (20).

Interviews and/or focus groups were conducted in community locations and faculty facilities. The interview questions were translated into the local Bisayan language for community member interviews, with the data later translated back into English. The data was recorded electronically for all participants, and transcription performed by the research team. After transcription, the original digital recordings (*via* mobile phone app or apple voice memos) were re-analysed for confirmation of content to cross-check with the data collector and researchers to clarify comments where necessary, and to achieve group consensus on key understandings from the data. All gathered data in various forms was stored in a secure filing cabinet, while digital data collected were coded and secured in a password protected database.

### Data Analysis

While the data was collected by a local team of ADZU-SOM researchers, the analysis was undertaken in partnership with a team of external THEnet-associated researchers with expertise in social accountability. To address internal validity, a matrix template for thematic analysis was developed using Gale et al's framework method for the analysis of qualitative data in multi-disciplinary health research (21), with an inductive approach then used to code and categorise the data into themes (20). The analysis of the interview and focus group questions was conducted locally and checked by two authors for investigator triangulation; differences were resolved through discussion. The team systematically reviewed the themes, and organized the evidence of student and graduate impacts on the general community using the key domains of THEnet's social-accountability framework (13, 14), while evidence of health service delivery impacts were organized using the WHO's “6 Building Blocks of Quality Health Systems” framework (3). This multi-faceted approach allowed interview data to be triangulated across the different participant groups and across the range of impact areas. To further ensure trustworthiness of the data, two authors not involved in the coding process then audited the analytic matrix, choice of quotes, and the thematic analysis, followed by asking an external educational expert to verify the validity of all analyses.

## Results

### Philosophy and Values of the School

ADZU-SOM faculty were fully committed to and focused on realising their vision of addressing the health workforce needs of Zamboanga Peninsula. “*A group of doctors realized the need for physicians who would help in solving the many health problems of the region. The shortage of doctors was so evident during the time that an intervention was an immediate necessity.”* The School's vision of bridging the health inequity gap in the region informs its mission and core values, and is accomplished through inter-sectoral partnerships with various stakeholders including the Department of Health (DOH), local government, and community stakeholders. Social accountability attributes that inform the School's values are: equity, quality, relevance, cost effectiveness social justice, community engagement and partnership, cultural sensitivity, mutual transformation, access to education, altruism, and responsiveness to community needs. “*Another factor that enabled the operation of a medical school is the faculty members' strong inclination to serve the society. During its founding years, all faculty believed in [the] vision of the school and members committed to teach pro bono…”* These values have been integrated into the governance and management of the School.

### Transformational Learning

#### Curriculum Content

The school's curriculum was developed through a region-wide consultative process with key stakeholders. The most common regional and national causes of morbidity and mortality formed the core content of the curriculum. Two students commented:

“*The curriculum has a great impact; not just attitude toward me but also on how I deal with other people. Personally when I got in the medical school, I had no idea that this was a community oriented school. This school changed my attitude and now I'm aware what my community looks like …. I did not [know] that there are types of community that really needs medical students to help them, inspire them to have a better community*.”

“*The school's curriculum helped me change my perspective in community service and caring [for the underserved]. At first, I was just looking at community services as tiresome, and going to the community as something similar to a medical mission. But with the help of the school's curriculum, I was able to look at the situation in a holistic approach*.”

#### Community-Engaged Learning and Development

ADZU-SOM's community-engaged curriculum is one of its unique aspects. Through this immersion program, the ADZU-SOM accomplishes its vision of “experiential learning” through community service – one of the core values of the ADZU-SOM. Overall, students spend close to 50% of the 4-year program based in the community. To internalise this value, students are completely immersed in the community to actively enhance engagement with stakeholders in promoting public health through joint research projects and activities, with the ultimate goal being students staying and practicing in the community after graduation.

A graduate commented that placement in communities and service learning contribute to community development: “*During our community exposure, we were asked to develop a CHP* [Community Health Plan]. *My duties as MHO (Municipal Health Officer) are quite similar to my community immersion. In many ways, the community immersion prepared me for this job*.”

Another graduate commented, “… *with the community exposures we had we were trained and we were able to understand the whole process from assessment, planning, which agencies to approach and coordinate with, whom to ask for help, and then we were tasked to implement and evaluate the process*.”

Final year students also had similar opinions, but admitted that developing a CHP was not an easy task. One student mentioned it was “*difficult to implement community health programs but then it is still possible given the skills and knowledge we have acquired*.”

### SAHPE Contribution to Health System Strengthening

#### Health Workforce

The ADZU-SOM's strong focus on community-engaged medical education which provides training where students learn rural health competencies through transformational learning and service-learning has contributed to the retention of graduates in underserved areas. A graduate shared “*Initially it was not my ambition to be a doctor, this changed during my community exposures. I learned so many things, initially, I thought I was going to change the community, however, it was the community that changed me, it changed my purpose, perception, and beliefs about being a doctor. I am here not merely for the monetary gain but to serve. I am now employed and serve in the same community where I was assigned as a medical student.”*

Furthermore, increasing graduate retention strengthens the health system by providing increased access to physicians and to basic health services. (Summarized in **Table 2**).

To date, the ADZU-SOM has produced a total of 425 board-certified physicians since it began. Out of the total, 96% practice in the Philippines, with 63% practicing in the Zamboanga region, 39% in rural and remote areas, and 11% in armed conflict areas. This may have contributed to the data reported by the Philippine Statistics Authority between 2003 to 2017 showing significant improvements in the proportion of births attended by skilled health personnel by 38.4–71.1% in the Zamboanga Peninsula.

#### Health Service Delivery

The impact of graduates and students from ADZU-SOM on strengthening the health system was evidenced by community health professionals, who described many instances of positive change in local health systems. Examples of positive change included the development of a referral system, increases in health workforce resulting in increased service utilization, increases in out-patient consultations, and more births occurring in health facilities rather than elsewhere (summarized in Table 1). Including local, regional, and national health priorities as the core content of the curriculum ensures that the services provided to the community are relevant and essential.

**Table 1:** AdZU-SOM student and graduate impacts on local health service delivery using the WHO's “6 Building Blocks for Quality Health Systems” framework (3).

**WHO six building blocks of health system impact areas**	**Sample quotes**	**Improvement in health indices**
**A. HEALTH WORKFORCE**		
**Increased health workforce**		
Employment of qualified physicians in remote areas	*We lived in poor, underserved areas; for the past four years, I've been working in Alicia, and [sic] Molave, and Kabasalan; I can put to practice what (I learned) when I was a medical student*.	Empathy with community stakeholders
	*We are able to customize programs and identify their problems and then we are able to bring applicable programs and interventions there, similar to what was previously said, we are more on the promotion and prevention [programs] that is fit to a specific area*	Health services coverage extension
	*Pangutaran(Island) is three hours away from Jolo, Sulu (Province) by boat. Prior to my assignment, the people did not have a physician. Now they don't have to travel to access health services*.	
**B. HEALTH SERVICE DELIVERY**		
**Building functional health facilities**	“*They facilitated improvement and renovation of our health center”*	Infrastructure development
**Development of additional health services**		
1. Procurement of funds for hospitals and health centers. Pre-natal services and immunization	*Brought health services to the islets; it's like a mobile clinic where we did medical consults, vaccination, prenatal consult, health teaching circumcision; minor OR, if possible.”*	Health services coverage extension
2. Providing health services to remote areas *via* mobile clinics. Lying-in services/new born care	“*The RHU [Regional Health Unit was transformed] from OPD to 24 h service– to maximize services: 24 h lying in, Newborn Care. Medical students go on duty.”*	Health services coverage extension
3. TB DOTS program	“*They will look into our assigned area and joined us in examining patients under the NTP [National Tuberculosis Program].”*	Health services coverage extension
	“*There were many TB cases here before but now, there are no new cases.”*	
4. Issuing of death certificates in remotes areas	“*For instance, for medico-legal cases, where death certification is needed people will have to travel to Pagadian and spend 180 Php just to get a signature. Now we can issue it from here*.”	Health services coverage extension
5.24 h availability of primary healthcare services	“*. as much as possible we offer 24/7 service to cater the needs of the community.”*	Health services coverage extension
6. Smoking cessation counselling program	*We had meetings with the medical students on smoking cessation, alcohol drinking, and other vices*.	Community health program development
**C. ACCESS TO ESSENTIAL MEDICINES**		
**Provision of medical equipment and supplies**		
Provision of free medicine and contraceptive implants	“*They provided us complete sets of medical equipments for the health station including medicines, towels and scissors. The medicines are replenished to have continued supply.”*	Increased in medical facilities and supplies
**D. LEADERSHIP & GOVERNANCE**		
**Partnerships**		
Collaboration with local leadership and other stakeholders for the institution of health-related programs	“*I was able to help them. If they needed something from the community we were also there to support them. We helped them in the feeding program/cooking show [demonstration], we supported in the activities and work to be done.”*	Improved partnerships with local leaders
	“*They [Medical Students] also joined Municipal Council sessions and brought up concerns and needs like waste segregration and disposal, toilet sanitation. And they are also open to suggestions*.	
**Relationships**		
Good rapport with local leadership and non-government organizations	*Apart from the fact that we have gone to a place where few if not no doctors have gone like in the farflung areas. We have help the community learn what their full potential as a community and what resources they might have use for the community to expand their resources and to become a better community*.	Improved partnerships with local leaders
	*[The medical students] are industrious; here in the rural community, they endure it just to reach the children who needs help*.	
**Creation of local community policies on health**	*There is a solid waste management in [barangay] Mirangan, parents were encouraged to segregate biodegradable and non-biodegredable waste because of the local policy. Penalties were imposed especially to 4Ps members*.	Improved local accountability through policies and reinforcement of policies
**E. FINANCING**		
Procurement of funds for hospitals and health centers. Pre-natal services and immunization	“*Brought health services to the islets; it's like a mobile clinic where we did medical consults, vaccination, prenatal consult, health teaching circumcision; minor OR, if possible.”*	Increase budget allocation/raising funds for health
Applied automatic enrolment to the National Health Insurance program for indigent patients	“*We practice no balance billing in the hospital.”*	Improved financial risk protection and coverage for vulnerable population
**F. HEALTH INFORMATION SYSTEM**		
**Regular collection of community data on health**	“*…assessing, monitoring, and recording patients under the NTP [National Tuberculosis Program].”*	Timely recording and reporting of health data
	“*We did house to house survey in search for malnourished children, we recorded their weight monitored them, and fed them.”*	
	“*We were able to gather baseline data that can be utilized by the barangay and the local government, I think that is a benefit that the barangay* [village/small local community] *got when we were assigned there*.”	

It was also noted by health professionals in local facilities that ADZU-SOM graduates and students had good communication skills and were approachable and confident in dealing with patients. The many community-based health promotion projects and increased infrastructure provided through advocacy from the graduates and students were also valued. Many of these health service delivery system strengthening activities also have system-wide effects that encompass the WHO “6 Building Blocks of quality health systems” (3).

#### Access to Essential Medicines

Through the community engagement program embedded in the curriculum, medical students collaboratively work with the community to provide various health services and essential medicines. A barangay health worker mentioned “*They provided our complete sets of medical equipments for the health station including medicines, towels, and scissors. The medicines are replenished to have continued supply.”* Providing access to essential medicines especially to the underserved population decreases out-of-pocket expenditures and contributes to better health outcomes.

#### Leadership and Governance

The WHO states that governance and leadership include ensuring strategic policy frameworks exist and are combined with effective oversight, coalition-building, regulation, attention to system design, and accountability (3). The ADZU-SOM contributes to strengthening the health system through leadership and governance by collaborating with local community leaders and stakeholders in identifying and seeking solutions to address the problems in their respective communities. Several projects implemented in the community involve supporting local leaders in drafting local community policies to reinforce existing laws and ensure the sustainability of programs and projects.

#### Financing

To strengthen health systems, funds should be sufficiently appropriated for health without the risk of financial hardship (3). The ADZU-SOM students through the community engagements work with the local leaders to collaborate with government and non-government agencies to raise money to fund local projects that include building infrastructures and procurement of essential equipments.

#### Health Information System

Collection of accurate health information is needed to create evidence-based interventions as well as to ensure that these interventions are effective. Together with community residents and volunteers, students gather health and health-related community data (such as social information and social determinants of health data) early in their community immersions. This data serves as a guide for the community in identifying needs and is used in the creation of programs to address the identified needs.

### Community Impact - Meeting Health Needs Through Research Intervention Studies

Community leaders, community health workers, and community members also confirmed ADZU-SOM graduates and students had a significant impact on their communities through their public health action research and community development projects (summarised in Table 2).

**Table 2:** ADZU-SOM student and graduate impacts on the general community using Training for Health Equity Network's (THEnet) Social Accountability Framework (13, 14).

**Public health action research and community development**		
**Impact areas**	**Actual quotes (in translation)**	
	**Community health workers**	**Community leaders/members**
**Health promotion projects**		
1. Hypertension	*We have a schedule, we conduct health teachings on diabetics and hypertensive patients*	*[The medical students] helped me a lot especially on hypertension, we exercised. Prizes were given to encourage [participation among] community members and senior citizens*.
2. Immunization	*[Medical students] conducted health teachings on. immunization of children*.	*Parents were well-informed on topics like immunizations and check ups*
3. Childhood nutrition	*[The medical students] taught and gave information on follow up check-ups and ways to address malnutrition*.	*[The medical students] taught us proper nutrition*.
4. Breastfeeding	*The medical students] taught mothers how to breastfeed*.	
5. Sanitation	*[The medical students] conducted health teachings on. environment and sanitation*.	*There is a great decline now-a-days in dengue since people are more knowledgeable on sanitation and eradication of mosquito breeding places*.
**Provided leadership for community development projects and Infrastructure development**		
1. Toilet construction facilitation	*[The medical students] constructed a toilet; toilets are important so that children will not get sick. They were able to construct the toilets with the help of the community people and the barangay council*.	*.households were provided with sanitary toilets as one of the students' [health] project as well as teachings [on sanitation/cleanliness]*
2. Construction of projects	*[The medical students] initiated construction of a freedom stage*.	*[The medical students] can help the community, they can make projects. There was a time, they made [facilitated] the construction of a bridge over a river; like now, it is raining [hard], the water level goes up, people can still cross the river.The health center was in bad condition. [The medical students] contributed to repair it through carpentry work and painting*.
3. Community livelihood projects	*Vermiculture and vegetable gardening managed by the barangay council*	*Livelihood projects on mat-making and nata de coco still continue to exist*.
4. Solid waste management	*[The medical students] taught us a lot especially on solid waste management, biodegradable and non-biodegradable segregation*.	*[The medical students] made a big impact. I learned important information on health and waste disposal*.

In particular, community leaders appreciated ADZU-SOM's strategy of community immersion. According to a local leader, the ADZU-SOM “*is the only medical school which extended its services here in Zamboanga Del Sur. It was with great joy that I was asked to prepare three areas for the medical students because they can help a lot. The chosen barangays* [village/small local community] *were so happy to receive them because there were students to guide them on health. ADZU-SOM brought joy and help. … Ateneo de Zamboanga University School of Medicine is the only school that reached out here*.” In a separate interview, another local leader echoed this appreciation: “*They [students and graduates] contributed a lot for the welfare of the patients.”*

The data suggests that ADZU-SOM accomplishes its vision through instilling community-oriented values in students as a result of service learning and community-engaged immersion. The following student quotes capture this:

“*The school's curriculum and the impact of community service was life changing. Before I entered medical school, I am a kind of person that I only care about myself and my family, I don't care about the community…an introvert kind of person. But when I got to medical school, life doesn't revolve around in just four corners. I saw the bigger picture. It was life changing*.”

A graduate working as a Municipal Health Officer (MHO), shared a similar experience: “*The community exposure [has impact on the attitude toward the community] because when you are in the hospital your focus is on the disease but when you are in the community, you also consider why the disease exists, why the referral was late – some factors not directly related to health, social factors that come into it. You can see a much bigger view*.”

## Discussion

This case study contributes to the understanding of SAHPE in the Philippines context, and describes qualitative evidence to support previous quantitative evidence of ADZU-SOM students and graduates having an impact on local health services and communities (10–12). Three key factors emerged as contributing to the SAHPE at ADZU-SOM: (1) the faculty commitment to the school's vision and their ability to operationalise its values, (2) a transformative, community-engaged, experiential learning approach, and (3) students and graduates contributing to a skilled local health workforce and provision of more services. The findings suggest that ADZU-SOM accomplishes its vision through instilling community-oriented values in its graduates and students as a result of service learning and community-engaged immersion.

The findings strongly supports and explains quantitative studies describing impacts of ADZU-SOM students and graduates on local health systems (10–12). Many examples of strengthening “service delivery” (Table 1) were given by community leaders and health workers across all the WHO's recommended “6 Building Blocks of quality health systems” (3). In addition to these health service actions, there was much evidence (Table 2) of ADZU-SOM students and graduates having impact on patients and members of the general community by promoting their use of healthcare services, and their knowledge and use of health protective and health promoting behaviours.

The 2013 WHO World Health Report (1) states “the goal of universal health coverage is to ensure that all people obtain the health services they need – prevention, promotion, treatment, rehabilitation and palliation.” The findings in Tables 1, 2 describe student-led activities in treatment, prevention, promotion, and health infrastructure activities undertaken in ADZU-SOM's final “internship” year in rural communities – communities that are specifically chosen for having the greatest health needs. The benefits from having a significant number of treatment, prevention and health promotion activities occurring simultaneously in areas of great need and delivered by a significant student workforce trained in public health, community development and the key health issues of that community, cannot be understated.

While the extra investment of running a socially-accountable, community-based medical training program can be considered well-balanced by the positive community health outcomes resulting from students' and graduates' public health and health infrastructure projects, implementing community-based medical training has its challenges (22). Resistance from governing bodies, health professional education sectors and especially conventional medical schools was experienced toward ADZU-SOM's innovative curriculum involving intensive community engagement; this resistance delayed the school's achievement of national accreditation. Permission to operate was only granted through constant dialogue, discussions, and lobbying with regional and national stakeholders. In addition, the paradigm shift inherent in a community-based program demanded retraining of ADZU-SOM faculty; with many clinicans having no formal training in teaching. The ADZU-SOM had to create faculty development programs to address these demands in in collaboration with local and national partner institutions and networks, to address these demands. Lastly, the admission criteria of preferentially selecting students coming from low-income bracket families needs constant financial support. For example, the average cost of one student per semester is P172,000 (~US$3,440), and the average price associated with running the community engagement program per year is P900,000 (~US$18,000). To support these core curriculum components, ADZU-SOM has had to continuously challenge and recruit local benefactors to invest in the medical program.

As a major positive of ADZU-SOM's service learning model involving extensive community placements, students participate in community development projects throughout their training, as well as receive a good understanding of the key health issues in local rural communities. This helps students consolidate their learnings of how public health and the social determinants influence the health of individuals and the overall community. In addition, students contribute to community development by implementation of their own community health plan involving health promotion, intervention and rehabilitation activities developed collaboratively with community members. From Year 1, ADZU students are assigned to a community where they will do a significant portion of their training; returning to the very same community during the entirety of their course. This strategy strengthens ties between students and community members, and gives students greater community exposure and repeated opportunities to understand the importance of their work and local cultures.

Given that improving both coverage of services and access of all community members to health services across all communities lies at the heart of the WHO health system strengthening strategy, the study findings support previous evidence that key health system factors are being enhanced across Zamboanga Peninsula by ADZU-SOM students and graduates. The study findings show that ADZU-SOM students and graduates have dramatically broadened the range of health services (programmes, interventions, goods) to local communities, and have extended access of these to wider population groups; thus contributing to the concept of universal access to health benefits. These findings also highlight how partnerships between health professional education institutions, communities, and local health systems can contribute to **social equity** – as there can be no health equity without social equity.

## Limitations

ADZU-SOM faculty conducted the focus group discussions, interviews, and report auditing; there were no external evaluators present in the sessions. While the authors feel this was not problematic for this study, this does present opportunity for bias. However, the internal evaluator was experienced in qualitative methods, and offered strength in terms of relevant understanding of local contexts, while triangulation with researchers external to the school minimised potential investigator bias.

There were also challenges translating from English to Bisayan and other local dialects. However, as the Filipino language does not always have the same meaning in English due to regional linguistic contexts, the internal evaluator employed a local person to check the analysis and conclusions. This approach also provided more data richness and a more balanced view of the situation; thus further enhancing the validity of this project's conclusions.

While individuals were selected for interviews based on their experiences, expertise and job position, it cannot be assumed they represent the opinions of the whole group. Similarly, the authors also acknowledge that the findings of this study may not apply directly to other community-engaged medical programs, although it is expected that the key principles would be similar.

## Conclusions

ADZU-SOM is an innovative medical school whose curriculum has pioneered an innovative approach to their challenging environment – long before the concept of social accountability was defined – by combining competency and problem-based instruction with experiential learning in the community, and having a curriculum that is responsive to both the needs of communities and sensitive to the social and cultural realities of Zamboanga Peninsula. ADZU-SOM has educated many students to become competent, fit-for-purpose doctors who now comprise the bulk of the medical workforce in Zamboanga Peninsula and surrounding island provinces. In addition, final year medical students also constitute a significant component of the health service delivery in communities served by ADZU-SOM. The findings suggest this community-based student workforce is having noticeable impacts due to a number of factors, including student motivation for community service, and training in public health and the social determinants of health.

The study findings suggest that ADZU-SOM has managed to evolve a consciousness toward community service among its final year students and graduates, adding evidence to ADZU-SOM's assertion that it is a fully socially-accountable health institution. This evolution, however, may not be solely ascribed to its curriculum, but also to the commitment of faculty and to the school's vision of working in partnership with local communities to bridge the health gap in Zamboanga Peninsula and neighbouring regions.

The findings also suggest ADZU-SOM students and graduates are strengthening local health systems across the WHO's recommended “6 Building Blocks of quality health systems” by developing health infrastructure and providing health education, health promotion and disease prevention activities accessible to all population groups; thus fulfilling the concept of universal access to health resources.

Overall, these findings provides empirical evidence that SAHPE schools can produce students and graduates capable of implementing key WHO health system strengthening strategies that lead to significantly improved health services and health equity in medically underserved areas, and over time, to achieving the sustainable development goals and Universal Health Coverage.

## Data Availability Statement

The raw data supporting the conclusions of this article will be made available by the authors, without undue reservation. Data may be made available upon request.

## Ethics Statement

This study involving human participants was reviewed and approved by the ADZU-SOM Ethics Review Committee and the Flinders University Human Research Ethics Committee (#7042) in Australia. All participants provided their written informed consent to take part in this study.

## Author Contributions

All authors of this manuscript have contributed substantially to the conception and design of the study, the acquisition, analysis and interpretation of data, drafting and revising the manuscript critically, and giving final approval of this version to be published.

## Conflict of Interest

The authors declare that the research was conducted in the absence of any commercial or financial relationships that could be construed as a potential conflict of interest.

## Figures and Tables

**Table:** 

**No. item**	**Guide questions/description**	**Reported on Page #**
**Domain 1: Research team and reflexivity**		
*Personal Characteristics*		
1. Interviewer/facilitator	Which author/s conducted the interview or focus group?	Halili, S.
2. Credentials	What were the researcher's credentials? E.g., PhD, MD	Halili, S. PhD Woolley, T. PhD Ross, S. MDR Reeve, C. PhD Neusy, AJ DTM&H Cristobal, F. MD-MHPEd Guingona, M. MD-MPH
3. Occupation	What was their occupation at the time of the study?	Halili, S. Dean of Graduate Studies AdZU-SOM Woolley, T. Evaluation Coordinator, JCU Ross, S. Project Manager, THENet Reeve, C. Rural General practitioner-JCU Neusy, AJ-Senior Director, Co-founder THENet Cristobal, F. Dean AdZU SOM Guingona, M. Faculty, AdZU SOM
4. Gender	Was the researcher male or female?	Male and Female
5. Experience and training	What experience or training did the researcher have?	Halili, S- Fulbright Research Fellow and FAIMER Fellow
*Relationship with participants*		
6. Relationship established	Was a relationship established prior to study commencement?	Halili, S. is an active faculty of the AdZU-SOM and provides mentorship and consultation in research
7. Participant knowledge of the interviewer	What did the participants know about the researcher? e.g., personal goals, reasons for doing the research	Written consent was obtained prior to the data collection, with the consent form also indicating the objectives and purpose of the research.
8. Interviewer characteristics	What characteristics were reported about the inter viewer/facilitator? e.g., Bias, assumptions, reasons and interests in the research topic	Limitations, page 11
**Domain 2: study design**		
*Theoretical framework*		
9. Methodological orientation and Theory	What methodological orientation was stated to underpin the study? e.g., grounded theory, discourse analysis, ethnography, phenomenology, content analysis	Methodology, study design, page 6
*Participant selection*		
10. Sampling	How were participants selected? e.g., purposive, convenience, consecutive, snowball	Methodology, data collection, page 6
11. Method of approach	How were participants approached? e.g., face-to-face, telephone, mail, email	Face-to-face, methodology, data collection, page 6
12. Sample size	How many participants were in the study?	Methodology, data collection, page 6
13. Non-participation	How many people refused to participate or dropped out? Reasons?	N/A
*Setting*		
14. Setting of data collection	Where was the data collected? e.g., home, clinic, workplace	Methodology, data collection, page 6
15. Presence of non-participants	Was anyone else present besides the participants and researchers?	Fellow student facilitating FGD, methodology, page 6
16. Description of sample	What are the important characteristics of the sample? e.g., demographic data, date	Methodology, data collection, page 6
*Data collection*		
17. Interview guide	Were questions, prompts, guides provided by the authors? Was it pilot tested?	Methodology, data collection, page 6
18. Repeat interviews	Were repeat interviews carried out? If yes, how many?	Methodology, data collection, page 6
19. Audio/visual recording	Did the research use audio or visual recording to collect the data?	Methodology, data collection, page 6
20. Field notes	Were field notes made during and/or after the interview or focus group?	N/A
21. Duration	What was the duration of the interviews or focus group?	1 h 30 min–2 h
22. Data saturation	Was data saturation discussed?	Methodology, data collection, page 6
23. Transcripts returned	Were transcripts returned to participants for comment and/or correction?	Methodology, data collection, page 6
**Domain 3: analysis and findings**		
*Data analysis*		
24. Number of data coders	How many data coders coded the data?	Three data coders
25. Description of the coding tree	Did authors provide a description of the coding tree?	Methodology-Data Analysis, page 6
26. Derivation of themes	Were themes identified in advance or derived from the data?	Methodology-Data Analysis, page 6
27. Software	What software, if applicable, was used to manage the data?	NVivo
28. Participant checking	Did participants provide feedback on the findings?	Methodology, data collection, page 6
*Reporting*		
29. Quotations presented	Were participant quotations presented to illustrate the themes/findings? Was each quotation identified? e.g., participant number	Results, page 7–10
30. Data and findings consistent	Was there consistency between the data presented and the findings?	Yes
31. Clarity of major themes	Were major themes clearly presented in the findings?	Yes
32. Clarity of minor themes	Is there a description of diverse cases or discussion of minor themes?	N/A

## Abbreviations

ADZU-SOM: Ateneo de Zamboanga University-School of Medicine
CHP: Community Health Plan
DOH: Department of Health
MHO: Municipal Health Officer
MD-MPH: Medicine and Public Health
SAHPE: Socially Accountable Health Professional Education
THEnet: Training for Health Equity Network
UHC: Universal Health Care.

## Appendix

**Consolidated criteria for reporting qualitative studies (COREQ): 32-item checklist**

Developed from: Tong A, Sainsbury P, Craig J. Consolidated criteria for reporting qualitative research (COREQ): a 32-item checklist for interviews and focus groups. *International Journal for Quality in Health Care*. 2007. Volume 19, Number 6: pp. 349–357.

**You must provide a response for all items. Enter N/A if not applicable**
